# Insulin resistance influences the impact of hypertension on left ventricular diastolic dysfunction in a community sample

**DOI:** 10.1002/clc.23145

**Published:** 2019-01-14

**Authors:** Adamu J. Bamaiyi, Angela J. Woodiwiss, Vernice Peterson, Monica Gomes, Carlos D. Libhaber, Pinhas Sareli, Gavin R. Norton

**Affiliations:** ^1^ Cardiovascular Pathophysiology and Genomics Research Unit, School of Physiology, Faculty of Health Sciences University of the Witwatersrand Johannesburg South Africa

**Keywords:** hypertension, insulin resistance, left ventricular diastolic function, obesity

## Abstract

**Background:**

Although obesity‐associated metabolic abnormalities (insulin resistance‐IR) may not play as marked a role in determining left ventricular (LV) diastolic dysfunction (DD) as hypertension, the impact of combinations of these risk factors on DD is unknown.

**Hypothesis:**

We hypothesized that IR influences the impact of hypertension on DD.

**Methods:**

In 704 randomly selected participants from a community sample with a high prevalence of hypertension (50.6%) and obesity (46.5%), we determined adiposity indices, IR from the homeostasis model (HOMA‐IR) and LV diastolic function using standard echocardiographic techniques.

**Results:**

HOMA‐IR was independently associated with lateral wall e' and E/e' (*P* < 0.05 to *P* < 0.005) as well as a diagnosis of DD (*P* < 0.02). Importantly, however, an enhanced relationship between HOMA‐IR and E/e' in hypertensives (n = 356, partial *r* = 0.15, *P* < 0.005) as compared to normotensives (n = 348, partial *r* = 0.02 *P* = 0.75) was noted. Consequently, as compared to normotensives, with adjustments for confounders, hypertension was independently associated with DD only in those with the highest tertile of HOMA‐IR (odds ratio = 2.65, 95% confidence interval = 1.29‐5.42, *P* < 0.01), while in those with the lowest tertile of HOMA‐IR, hypertension failed to show a higher prevalence of DD (*P* = 0.22).

**Conclusions:**

Insulin resistance enhances the impact of hypertension on LV DD. Thus, DD is more likely to occur with the combination of hypertension and IR.

## 1 INTRODUCTION

Heart failure with a preserved (normal) ejection fraction may contribute to close to half of all admissions for heart failure, and the outcomes may be equally as poor as heart failure with a reduced ejection fraction.^1^, ^2^, ^3^, ^4^ There is presently little evidence for proven treatment benefits for heart failure with a normal ejection fraction.^1^, ^5^, ^6^ Although diastolic dysfunction (DD) is central to the pathophysiology and outcomes of heart failure with a normal ejection fraction^7^, ^8^, ^9^, ^10^, ^11^ and pre‐clinical DD predicts the progression to heart failure with a normal ejection fraction,^12^ it is possible that the pathophysiological mechanisms responsible for DD may differ depending on the risk factors involved. In order to better identify therapeutic targets for heart failure with a preserved ejection fraction, an improved understanding of the role of the risk factors for DD is required. In this regard, the impact on DD of the combination of the commonly occurring co‐morbidities, hypertension, and obesity or the associated metabolic disturbances, is uncertain.

Some prior studies conducted in elderly populations, in patients referred for echocardiography, or in samples with a high proportion of participants receiving therapy, report on an equal or greater impact of obesity as compared to hypertension on left ventricular (LV) diastolic function.^13^, ^14^, ^15^ However, in studies conducted across the full adult age range in a community sample with a high proportion of obesity and hypertension, but a low proportion of participants who were receiving antihypertensive therapy, blood pressure (BP) was noted to be the main determinant of DD.^16^ Nevertheless, more recent evidence suggests that a more concentrically remodeled LV determines whether obesity‐related insulin resistance (IR) is associated with LV diastolic function.^17^ As hypertension is a strong determinant of concentric LV remodeling, the possibility exists that even if obesity or associated IR have only a modest impact on LV diastolic function, that obesity or its metabolic consequences may determine the extent to which DD occurs in hypertension. Consequently, in the present study, we aimed to determine whether adiposity indices or IR influence the extent to which DD occurs in hypertension in a community sample with a high prevalence of hypertension and obesity.

## 2 METHODS

### 2.1 Study sample

The present study was approved by the University of the Witwatersrand Committee for Research in Human Subjects (approval number M02‐04‐72 renewed as M07‐04‐69, M12‐04‐108, and M17‐04‐01). Participants gave informed, written consent. The study design has previously been described.^18^, ^19^, ^20^, ^21^ 1044 Participants of nuclear families of black African descent with siblings older than 16 years were randomly recruited from the South West Township of Johannesburg, South Africa for echocardiographic studies. Tissue Doppler measures of myocardial function were obtained in a sub‐study conducted in 704 participants from the time that these measures became routinely available.

### 2.2 Demographic and clinical information

A standardized questionnaire was administered to obtain demographic and clinical data.^18^, ^19^, ^20^, ^21^ Height, weight, and waist circumference (WC) were measured using standard approaches and participants were identified as being overweight if their body mass index (BMI) was ≥25 kg/m^2^, obese if their BMI was ≥30 kg/m^2^ and morbidly obese of their BMI was ≥35 kg/m^2^. Central obesity was defined as an enlarged WC (≥88 cm in women and ≥ 102 cm in men). Laboratory blood tests including percentage glycated hemoglobin (HbA1c) were performed. Fasting plasma insulin concentrations were determined from an insulin immulite, solid phase, two‐site chemiluminescent immunometric assay (Diagnostic Products Corporation, Los Angeles, California). Diabetes mellitus or an abnormal blood glucose control was defined as the use of insulin or oral hypoglycemic agents or a glycated hemoglobin (Roche Diagnostics, Mannheim, Germany) value greater than 6.5%. Insulin resistance was estimated by the homeostasis model assessment of IR (HOMA‐IR) using the formula (insulin [μU/mL] × glucose [mmol/L])/22.5.

Nurse‐derived conventional BP was measured according to guidelines using a mercury sphygmomanometer after 5 minutes of rest in the seated position as previously described.^20^ Five consecutive BP readings were obtained using an appropriately sized cuff, 30 to 60 seconds apart. The average of the five readings was taken as the BP. None of the visits had fewer than the planned BP recordings. Hypertension was defined as the use of antihypertensive medication or if the mean of the five conventional BP measurements was >140 (systolic BP) or 90 (diastolic BP) mm Hg in those not receiving medication.

### 2.3 Echocardiography

Echocardiographic measurements were performed as previously described^16^, ^17^, ^18^, ^19^ by two experienced observers (AJW and CDL) with the participants in the partial left decubitus position. All potential participants were assessed for mitral valve abnormalities as determined using two‐dimensional and color Doppler imaging and excluded if significant valve abnormalities were present. Left ventricular dimensions were determined using two‐dimensional directed M‐mode echocardiography in the short axis view and these recordings were analyzed according to the American Society of Echocardiography convention.^22^ The LV dimensions were measured only when appropriate visualization of both the right and the left septal surfaces occurred and where the endocardial surfaces of both the septal and posterior wall were clearly visible. Left ventricular ejection fraction was calculated using the biplane Simpson method. Left ventricular mass (LVM) was determined using a standard formula^23^ and indexed (LVMI) to height^1.7^.

Left ventricular diastolic function was assessed from a pulsed wave Doppler examination of the mitral inflow at rest and using tissue Doppler indices (TDI) as well as left atrial volumes (LAV).^24^ Pulse wave Doppler recordings of trans‐mitral velocity were obtained with the sample volume at the tip of the mitral valve in the apical four‐chamber view. Trans‐mitral velocity measurements were obtained during the early (E) period of left ventricular diastolic inflow. To perform TDI, the velocity of myocardial tissue lengthening at the level of the mitral annulus was recorded in the apical four‐chamber view. The sample volume was positioned at the septal and lateral corners of the mitral annulus. To determine diastolic function using TDI, peak velocities during early (e') diastole were measured. Data were expressed as the E/e' ratio (an index of LV filling pressures). Because mitral annular velocity (e') remains constant and trans‐mitral flow (E) increases with an increased filling pressure, E/e' ratio correlates well with left ventricular filling pressures. Left atrial volume indexed to body surface area, was calculated using the area‐length method, where length was defined as the shortest of the two long axes measured in the apical four‐chamber and two‐chamber views. Left atrial area was measured by planimetry in the apical four‐chamber and two‐chamber views at left ventricular end systole (maximum left atrial dimensions). As no participants had a reduced EF, LV DD was identified by the presence of at least two of the following: a lateral e' < 10 cm/s or a septal e' < 8 cm/s, E/e' > 14, or LAV index ≥34 mL/m^2^.^24^

### 2.4 Data analysis

Database management and statistical analyses were performed with SAS software, version 9.4 (SAS Institute Inc., Cary, North Carolina). Data from individuals were averaged and expressed as mean ± SD or the SE of the mean (SEM). To improve on the distribution of data, HOMA‐IR, lateral e', septal e', E/e', and LAV index were logarithmically transformed. To determine independent relations, multivariate adjusted linear (continuous data) or logistic (discrete data) regression analysis was performed. Indexes of diastolic LV function were adjusted for several confounders associated with diastolic function noted in bivariate analysis. Relationships (partial *r* values) were compared with z‐statistics.

## 3 RESULTS

### 3.1 Characteristics of study sample

Table 1 gives the demographic and clinical characteristics of the normotensive and hypertensive participants. More women than men participated in the study and a high proportion of participants, particularly, the hypertensives, were overweight, obese or morbidly obese and had central obesity. As compared to participants recruited prior to TDI becoming available, participants in whom echocardiography was performed once routine TDI became available, were modestly older with more abdominal obesity, but a lower HOMA‐IR and LVMI and more were receiving treatment for hypertension (Table S1, Supporting Information). A 5.5% of the normotensives and 25.0% of the hypertensives had DD and this was largely determined by a combination of either reductions in lateral or septal e' and increases in E/e' (75%). No participants had an ejection fraction <40% and 4.4% had an ejection fraction <50%. Of the sample 39.3% had LV hypertrophy (LVH) (LVMI > 80 g/m^1.7^ for men and >60 g/m^1.7^ for women) and 18.1% had concentric LV remodeling (relative wall thickness >0.42). A greater proportion of hypertensives than normotensives had LVH and concentric LV remodeling.

**Table 1:** Characteristics of the study sample

	All	Normotensives	Hypertensives
Sample number (% female)	704 (67.3%)	348 (65.8%)	356 (68.8%)
Age (years)	47.2 ± 18.1	37.8 ± 14.5	56.5 ± 15.5**
Body mass index (kg/m^2^)	30.1 ± 8.1	27.9 ± 8.0	32.2 ± 7.6**
% Overweight/obese/morbidly obese	23.0/20.2/26.3	27.0/16.1/16.1	19.1/24.2/36.2**
Waist circumference (WC) (cm)	93.9 ± 18.2	87.6 ± 17.2	100.0 ± 17.0**
% Abnormal WC	52.3	39.2	65.1**
Regular tobacco (% subjects)	16.5	18.1	14.9
Regular alcohol (% subjects)	19.5	21.8	17.1
% Diabetes mellitus or an HbA1c > 6.5%	14.4	5.7	22.7**
% Treated for hypertension	29.3	0	57.9**
HOMA‐IR	2.51 ± 3.92	2.13 ± 3.74	2.88 ± 4.07*
Brachial SBP/DBP (mm Hg)	128 ± 21/83 ± 13	114 ± 11/76 ± 8	141 ± 22/89 ± 13**
E/e'	7.5 ± 4.2	7.0 ± 3.3	9.2 ± 4.3**
Lateral e' (cm/s)	11.3 ± 4.1	13.2 ± 3.9	9.5 ± 3.4**
Septal e' (cm/s)	9.6 ± 3.6	11.2 ± 3.5	8.0 ± 3.0**
Left atrial volume (LAV) index (mL/m^2^)	19.7 ± 3.6	18.6 ± 7.0	20.9 ± 7.8**
Left ventricular (LV) mass index (g/m^1.7^)	62.7 ± 23.1	55.9 ± 20.3	69.2 ± 23.8**
LV relative wall thickness	0.36 ± 0.08	0.34 ± 0.07	0.38 ± 0.08**
% with diastolic dysfunction	15.3	5.5	25.0**

Abbreviations: DBP, diastolic blood pressure; e', myocardial tissue lengthening in early diastole at the mitral annulus; E/e', transmitral early blood flow velocity/velocity of the mean value of lateral and septal wall myocardial tissue lengthening in early diastole at the mitral annulus;HbA1c, glycated hemoglobin; HOMA‐IR, homeostasis model of insulin resistance; LV, left ventricle; SBP, systolic blood pressure .

* *P* < 0.01,

** *P* < 0.0001 vs normotensives.

### 3.2 Factors associated with LV diastolic function

With adjustments for confounders, systolic BP, and either WC, HOMA‐IR, or BMI were independently associated with lateral and septal wall e' and E/e' (Table S2). While WC and systolic BP were independently associated with LAV index, HOMA‐IR, and BMI were not (Table S2). However, with adjustments for confounders systolic BP and HOMA‐IR, but not WC or BMI were independently associated with the presence of LV DD (Table 2).

**Table 2:** Multivariate adjusted associations between risk factors and left ventricular (LV) diastolic dysfunction (DD) in a community sample (n = 704)

Models with	HOMA‐IR		Waist circumference (WC)		Body mass index (BMI)	
LV DD vs	Odds ratio (95% CI)	*P‐*value	Odds ratio (95% CI)	*P*‐value	Odds ratio (95% CI)	*P*‐value
HOMA‐IR/WC/BMI	1.428 (1.078‐1.890)	0.013	1.013 (0.995‐1.031)	0.16	1.039 (0.999‐1.080)	0.06
Age	1.038 (1.017‐1.059)	<0.0005	1.035 (1.015‐1.056)	<0.001	1.037 (1.017‐1.058)	<0.0005
SBP	1.021 (1.010‐1.033)	<0.0005	1.020 (1.008‐1.031)	<0.001	1.019 (1.008‐1.031)	0.001
Female gender	1.663 (0.863‐3.202)	0.13	1.319 (0.679‐2.563)	0.41	1.193 (0.590‐2.412)	0.62

Abbreviations: CI, confidence interval; HOMA‐IR, homeostasis model of insulin resistance; LV, left ventricle; SBP, systolic blood pressure.

Additional adjustors include regular tobacco use, regular alcohol consumption, treatment for hypertension, diabetes mellitus, and pulse rate.

### 3.3 Impact of IR on LV diastolic function in hypertensives and normotensives

**Figure 1:**
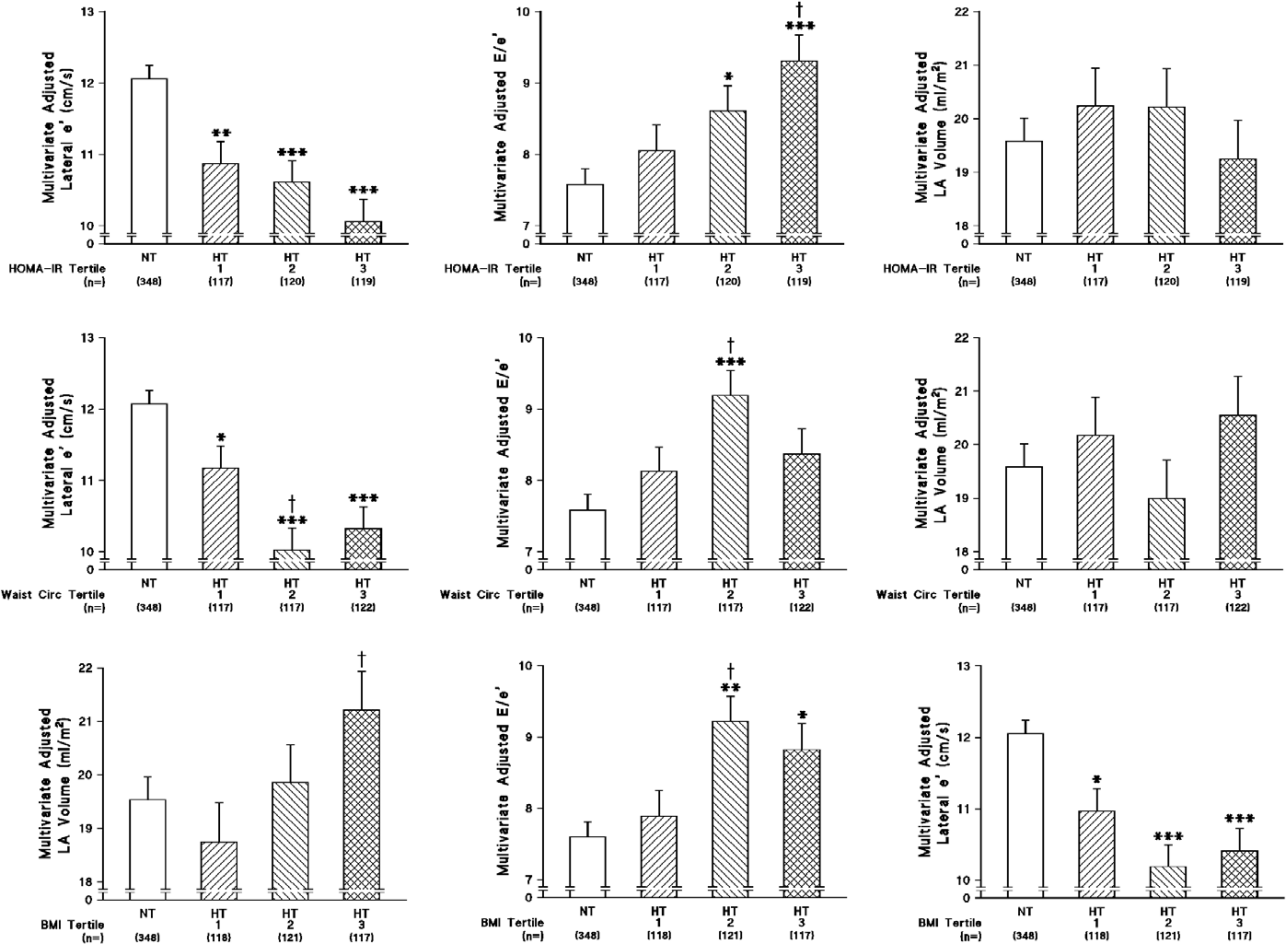
Multivariate adjusted indices of left ventricular diastolic function in normotensives and across tertiles of the homeostasis model of insulin resistance (HOMA‐IR) (upper panel), waist circumference (WC) (middle panel) or body mass index (BMI) (lower panel) in hypertensives of a community sample. Adjustments are for age, sex, systolic blood pressure, pulse rate, regular smoking, regular alcohol consumption, and diabetes mellitus. Tertiles of HOMA‐IR, WC, and BMI are defined Table S3. *P < 0.02, **P < 0.001, ***P < 0.0001 vs normotensives. †*P* < 0.05 vs hypertensives HOMA‐IR tertile 1 or vs hypertensives waist circumference tertile 1. e', myocardial tissue lengthening in early diastole at the mitral annulus; E/e', transmitral early blood flow velocity/velocity of the mean value of lateral and septal wall myocardial tissue lengthening in early diastole at the mitral annulus; LV, left ventricle

**Figure 2:**
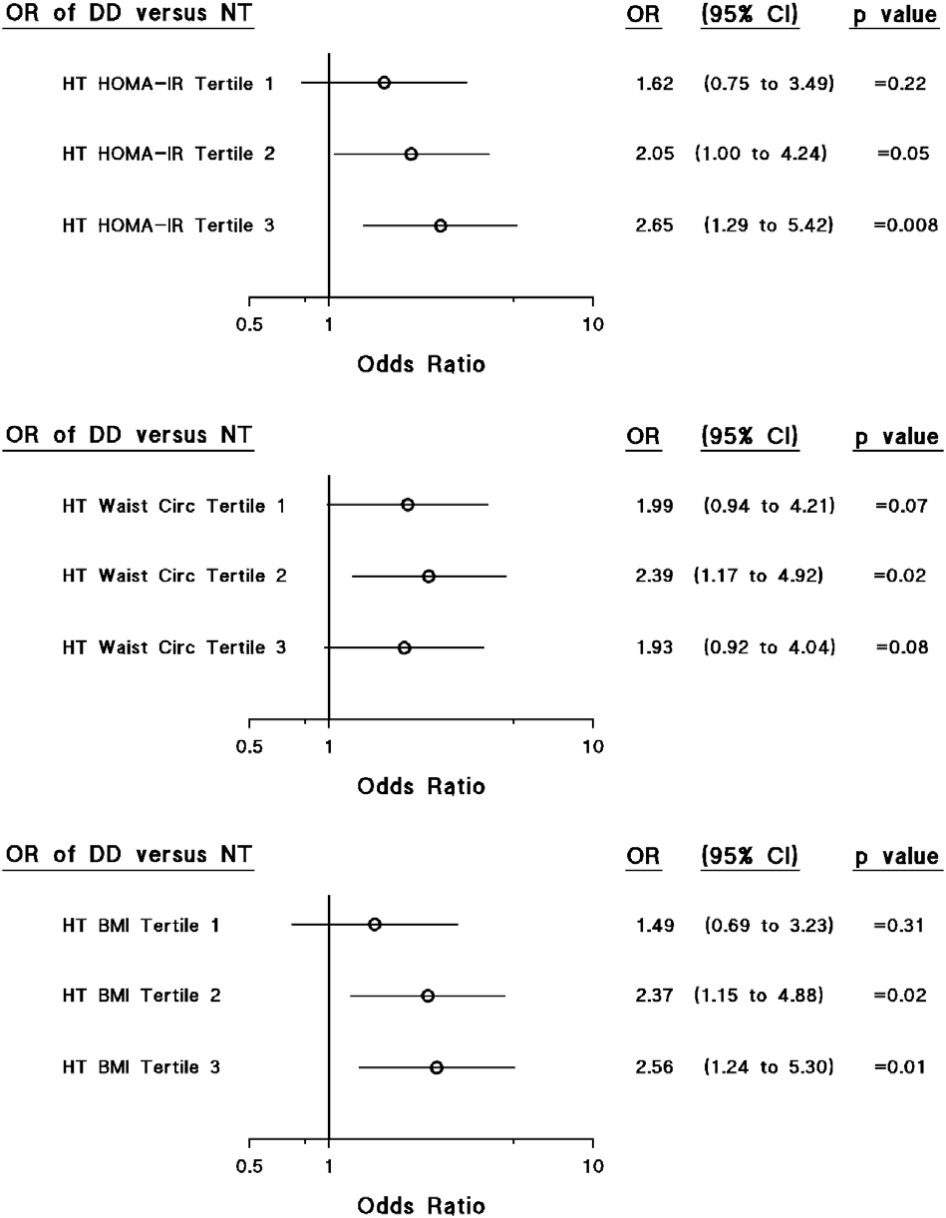
Impact of insulin resistance (homeostasis model‐homeostasis model of insulin resistance [HOMA‐IR]), waist circumference (WC) or body mass index (BMI) on the odds of independent associations between hypertension and left ventricular diastolic dysfunction. Adjustments in the left panels are for age, sex, pulse rate, regular smoking, regular alcohol consumption, and diabetes mellitus. Tertiles of HOMA‐IR, WC, and BMI are defined in Table S3

In both normotensives and hypertensives, WC (and BMI) as well as HOMA‐IR were independently associated with lateral wall e' (Table 3). However, in normotensives, but not in hypertensives, WC or BMI were independently associated with E/e', while in hypertensives, but not normotensives, HOMA‐IR was independently associated with E/e' (Table 3). Importantly, as compared to normotensives, this translated into an independent effect of hypertension on E/e' (and lateral wall e') only in those hypertensives with a HOMA‐IR in the upper two tertiles (Figure 1). Although hypertension was also only independently associated with an increased E/e' (and lateral wall e') in the upper two tertiles of BMI this effect failed to show a stepwise relationship and hypertension was also only independently associated with an increased E/e' (and lateral wall e') in the second tertile of WC (Figure 1). Although more hypertensives had DD across all tertiles of HOMA‐IR, WC, or BMI (Figure S1), these effects were largely attributed to age differences. Indeed, beyond age and other confounders hypertension was only independently associated with an increased odds of DD in those with the higher tertiles of HOMA‐IR, WC, or BMI (Figure 2). Importantly, a stepwise increase in the odds of DD occurred across tertiles of HOMA‐IR, but inconsistent effects were noted for WC and BMI (Figure 2).

**Table 3:** Impact of hypertension on multivariate adjusted relationships between the homeostasis model of insulin resistance (HOMA‐IR), waist circumference or body mass index and indexes of left ventricular diastolic function in a community sample (n = 704)

	Normotensives (n = 348)			Hypertensives (n = 356)		
	β‐Coefficient ± SEM	Partial *r* (95% CI)	*P*‐value	β‐Coefficient ± SEM	Partial *r* (95% CI)	*P*‐value
HOMA‐IR vs						
Log lateral wall e'	−0.018 ± 0.008	−0.12 (−0.23 to −0.02)	0.024	−0.017 ± 0.008	−0.11 (−0.21 to −0.002)	0.046
Log septal wall e'	−0.010 ± 0.008	−0.07 (−0.17 to 0.03)	0.19	−0.013 ± 0.008	−0.08 (−0.18 to 0.02)	0.13
Log E/e'	0.003 ± 0.011	0.02 (−0.09 to 0.12)	0.75	0.031 ± 0.010**	0.15 (0.05 to 0.25)	0.004
Left atrial (LA) volume index	0.009 ± 0.010	0.05 (−0.06 to 0.15)	0.39	0.006 ± 0.010	0.03 (−0.07 to 0.14)	0.57
Waist circumference vs						
Log lateral wall e'	−0.0018 ± 0.0004	−0.23 (−0.34 to −0.12)	<0.0001	−0.0011 ± 0.0005	−0.13 (−0.24 to −0.02)	0.020
Log septal wall e	−0.0021 ± 0.0006	−0.22 (−0.32 to −0.10)	<0.0005	−0.0013 ± 0.0005	−0.15 (−0.26 to −0.04)	0.008
Log E/e'	0.0023 ± 0.0006	0.22 (0.11 to 0.32)	<0.0005	0.0009 ± 0.0007	0.08 (−0.03 to 0.19)	0.16
LA volume index	0.0014 ± 0.0006	0.14 (0.03 to 0.25)	0.015	0.0004 ± 0.0006	0.004 (−0.11 to 0.12)	0.95
Body mass index vs						
Log lateral wall e'	−0.0032 ± 0.0009	−0.19 (−0.29 to −0.09)	<0.0005	−0.0017 ± 0.0011	−0.08 (−0.18 to 0.02)	0.13
Log septal wall e'	−0.0033 ± 0.0010	−0.17 (−0.27 to −0.07)	0.001	−0.0017 ± 0.0011	−0.08 (−0.18 to 0.02)	0.12
Log E/e'	0.0035 ± 0.0012	0.16 (0.05 to 0.26)	0.004	0.0023 ± 0.0014	0.09 (−0.02 to 0.19)	0.11
LA volume index	0.0010 ± 0.0012	0.05 (−0.06 to 0.15)	0.39	0.0024 ± 0.0013	0.10 (−0.01 to 0.21)	0.06

* Adjustments are for age, sex, systolic blood pressure, regular tobacco use, regular alcohol consumption, diabetes mellitus, and pulse rate.

** *P* < 0.05 vs β‐coefficient in normotensives.

## 4 DISCUSSION

The main findings of the present study are as follows: In a community sample with a high prevalence of obesity, indices of excess adiposity and IR were independently associated with indices of LV diastolic function (e' and E/e'). However, while HOMA‐IR was independently associated with lateral wall e' in both hypertensives and normotensives, HOMA‐IR was independently associated with E/e' in hypertensives, but not in normotensives. Consequently, HOMA‐IR determined whether hypertensives developed LV DD as compared to normotensives. In contrast, adiposity indices were associated with indices of diastolic function less well in hypertensives as compared to normotensives and adiposity indices were consequently not independently associated with DD.

Although several large studies have demonstrated that indices of excess adiposity are strongly and independently associated with indices of LV DD,^13^, ^14^, ^15^ these studies have been confounded by the use of predominantly elderly populations (where age is the principle determinant of DD), in select patients referred for echocardiography, or in samples with a high proportion of participants receiving antihypertensive therapy.^13^, ^14^, ^15^ In contrast, in an alternative study conducted in a much smaller cohort of the present community, but across the adult age range and in a sample with a high prevalence of obesity and hypertension, where antihypertensive therapy was employed in only half the hypertensives,^16^ BP was noted to contribute far more to DD than adiposity indices. In keeping with this prior study^16^ in the present study conducted in a much larger study sample of the same community we show that while BP and HOMA‐IR translated into DD, adiposity indices failed to do so. The ability of HOMA‐IR to associate with DD while adiposity indices did not, we attribute to an impact of HOMA‐IR, but not adiposity indices on E/e in the hypertensive, but not in the normotensive BP range. These data therefore suggest that IR is an important contributor to DD, but mainly in those with hypertension.

An important caveat of the present study is that the results do not suggest that IR mediates LV DD through hypertension. Indeed, relations between adiposity indices or HOMA‐IR and LV diastolic functional parameters were independent of systolic BP. Rather, the present study suggests that the impact of hypertension on relations between HOMA‐IR and DD should be viewed as an additive effect with systolic BP effects alone being more important, but with IR influencing whether hypertension translates into DD.

The criteria for the diagnosis of LV DD have been debated over several decades. As recently highlighted,^24^ in those with a normal ejection fraction, tissue Doppler indices of DD (e' and E/e), LAV index and the extent of tricuspid regurgitation, an index if pulmonary artery pressures, are recommended for the diagnosis of DD. Although we determined three of the four recommended measures of DD (lateral and septal wall e', E/e', and LAV index), at the time of initiating the present study, we did not determine the extent of tricuspid regurgitation. To diagnose DD, we nevertheless employed two of three criteria, while current guidelines recommend two of four (50%) criteria. Although there are no large studies that have demonstrated a relationship between adiposity indices and the extent of tricuspid regurgitation, it is therefore possible that we underestimated the prevalence of obesity‐associated DD in the present study. However, the present study is the first to assess relations between adiposity indices and DD determined according to contemporary guidelines,^24^ which do not include E/A if ejection fraction is within a normal range. In this regard, because of pseudo‐normalization of E/A, E/A is only recommended for use for the diagnosis of DD in those with a reduced ejection fraction. In this regard, the present and previous studies^13^, ^14^, ^15^ were conducted in participants with a largely normal ejection fraction. Hence, relations between HOMA‐IR or indices of excess adiposity and DD, as described in the present study, are more likely to reflect relations between an excess adiposity and actual DD than those previously described.^13^, ^14^, ^15^

An explanation for the impact of HOMA‐IR on E/e' (an index of LV filling pressures) and hence DD in hypertensives, but not normotensives in the present study, is unclear. It is possible that because hypertension is associated with concentric LV remodeling, and as recently demonstrated, a more concentrically remodeled LV determines the impact of IR on E/e',^17^ that IR only contributes to LV filling pressures when the LV is more concentrically remodeled. Importantly, however, the presence of hypertension in the present study did not influence the association between HOMA‐IR and lateral wall e', an index of LV relaxation, while previous work does show an impact of relative wall thickness on relations between HOMA‐IR and lateral wall e'.^17^ Hence, the impact of hypertension on relations between HOMA‐IR and E/e' may not be accounted for just by an effect of the extent of concentric LV remodeling, but by an alternative as yet unidentified factor.

There are several potential implications of the findings of the present study. First, in contrast to adiposity indices, which had less of an ability to determine the impact of hypertension on DD, the assessment of IR may better characterize hypertensives at risk of developing DD and hence heart failure with a preserved ejection fraction. In this regard, longitudinal studies are required to assess this question. Second, targeting IR as opposed to an excess adiposity per se with behavioral modification in hypertensives may have marked benefits to preventing the development of DD and hence heart failure with a preserved ejection fraction. In this regard, intervention studies are required to assess these hypotheses.

The limitations of the present study are as follows: First, this is a cross‐sectional study and hence, we cannot draw conclusions regarding causality. Whether the development of IR influences that of LV DD in those with hypertension rather than normotension therefore requires further study. Second, we are not statistically powered to perform sex‐specific analysis and hence, it is possible that as more women than men volunteered for the study, the results may relate mainly to women.

In conclusion, in a relatively large community‐based sample with a high prevalence of obesity and hypertension, we show that independent of confounders, the extent of IR influences whether hypertension translates into diastolic dysfunction. These data suggest that from a clinical perspective, hypertensives with IR may be particularly prone to the development of LV DD and thus possibly the progression to heart failure with a preserved ejection fraction. Consequently, targeting IR in hypertension may have marked benefits for preventing the development of heart failure.

## CONFLICTS OF INTEREST

The authors declare no potential conflict of interests.

## Supporting information


**FIGURE S1** Prevalence of left ventricular diastolic dysfunction (DD) in hypertensives as compared to normotensives across tertiles of the homeostasis model of insulin resistance (HOMA‐IR), waist circumference (WC) or body mass index (BMI) in the whole group and across similar age ranges. Tertiles of HOMA‐IR, WC, and BMI are defined in on‐line Table S3.

**TABLE S1** Characteristics of community sample without tissue Doppler imaging (TDI).
**TABLE S2** Relative contribution (standardized β‐coefficient) of the homeostasis model of insulin resistance, waist circumference or body mass index vs alternative risk factors toward indices of left ventricular diastolic function in a community sample (n = 704).
**TABLE S3** Ranges of tertiles of the homeostasis model of insulin resistance, waist circumference, and body mass index in normotensives and hypertensives.

## References

[1] Borlaug BA , Redfield MM . Diastolic and systolic heart failure are distinct phenotypes within the heart failure spectrum. Circulation. 2011;123:2006‐2014. 21555723 10.1161/CIRCULATIONAHA.110.954388 PMC3420141

[2] Lee DS , Gona P , Vasan RS , et al. Relation of disease pathogenesis and risk factors to heart failure with preserved or reduced ejection fraction: insights from the Framingham heart study of the National Heart, lung and blood institute. Circulation. 2009;119:3070‐3077. 19506115 10.1161/CIRCULATIONAHA.108.815944 PMC2775498

[3] Owan TE , Hodge DO , Herges RM , Jacobsen SJ , Roger VL , Redfield MM . Trends in prevalence and outcome of heart failure with preserved ejection fraction. N Engl J Med. 2006;355:251‐259. 16855265 10.1056/NEJMoa052256

[4] Bhatia RS , Tu JV , Lee DS , et al. Outcomes of heart failure with preserved ejection fraction in a population‐based study. N Engl J Med. 2006;355:260‐269. 16855266 10.1056/NEJMoa051530

[5] Borlaug BA , Paulus WJ . Heart failure with preserved ejection fraction: pathophysiology, diagnosis, and treatment. Eur Heart J. 2011;32:670‐679. 21138935 10.1093/eurheartj/ehq426 PMC3056204

[6] Pitt B , Pfeffer MA , Assmann SF , et al. TOPCAT investigators. Spironolactone for heart failure with preserved ejection fraction. N Engl J Med. 2014;370:1383‐1392. 24716680 10.1056/NEJMoa1313731

[7] Zile MR , Baicu CF , Gaasch WH . Diastolic heart failure: abnormalities in active relaxation and passive stiffness of the left ventricle. N Engl J Med. 2004;350:1953‐1959. 15128895 10.1056/NEJMoa032566

[8] Westermann D , Kasner M , Steendijk P , et al. Role of left ventricular stiffness in heart failure with normal ejection fraction. Circulation. 2008;117:2051‐2060. 18413502 10.1161/CIRCULATIONAHA.107.716886

[9] Burke MA , Katz DH , Beussink L , et al. Prognostic importance of pathophysiologic markers in patients with heart failure and preserved ejection fraction. Circ Heart Fail. 2014;7:288‐299. 24365774 10.1161/CIRCHEARTFAILURE.113.000854 PMC5947992

[10] Mohammed SF , Borlaug BA , Roger VL , et al. Comorbidity and ventricular and vascular structure and function in heart failure with preserved ejection fraction. A community‐based study. Circ Heart Fail. 2012;5:710‐719. 23076838 10.1161/CIRCHEARTFAILURE.112.968594 PMC3767407

[11] Shah AM , Claggett B , Sweitzer NK , et al. Cardiac structure and function and prognosis in heart failure with preserved ejection fraction. Findings from the echocardiographic study of the treatment of preserved cardiac function heart failure with an aldosterone antagonist (TOPCAT) trial. Circ Heart Fail. 2014;7:740‐751. 25122186 10.1161/CIRCHEARTFAILURE.114.001583 PMC4916914

[12] Wan S‐H , Vogel MW , Chen HH . Pre‐clinical diastolic dysfunction. J Am Coll Cardiol. 2014;63:407‐416. 24291270 10.1016/j.jacc.2013.10.063 PMC3934927

[13] Russo C , Jin Z , Homma S , et al. Effect of obesity and overweight on left ventricular diastolic function. A community‐based study in an elderly cohort. J Am Coll Cardiol. 2011;57:1368‐1374. 21414533 10.1016/j.jacc.2010.10.042 PMC3077126

[14] Çil H , Bulur S , Türker Y , et al. Impact of body mass index on left ventricular diastolic dysfunction. Echocardiography. 2012;29:647‐651. 22486526 10.1111/j.1540-8175.2012.01688.x

[15] Aljaroudi W , Halley C , Houghtaling P , et al. Impact of body mass index on diastolic function in patients with normal left ventricular ejection fraction. Nutr Diabetes. 2012;2:e39. 23448803 10.1038/nutd.2012.14 PMC3432184

[16] Millen AME , Libhaber CD , Majane OHI , et al. Relative impact of blood pressure as compared to an excess adiposity on left ventricular diastolic dysfunction in a community sample with a high prevalence of obesity. J Hypertens. 2014;32:2457‐2464. 25215435 10.1097/HJH.0000000000000330

[17] Peterson V , Norton GR , Raymond A , et al. Insulin resistance‐associated decreases in left ventricular diastolic function are strongly modified by the extent of concentric remodeling in a community sample. Int J Cardiol. 2016;220:349‐355. 27390954 10.1016/j.ijcard.2016.06.206

[18] Woodiwiss AJ , Libhaber CD , Majane OHI , Libhaber E , Maseko M , Norton GR . Obesity promotes left ventricular concentric rather than eccentric geometric remodeling and hypertrophy independent of blood pressure. Am J Hypertens. 2008;21:1144‐1151. 18756261 10.1038/ajh.2008.252

[19] Libhaber CD , Woodiwiss AJ , Booysen HL , et al. Differential relationships of systolic and diastolic blood pressure with components of left ventricular diastolic dysfunction. J Hypertens. 2014;32:912‐920. 24509119 10.1097/HJH.0000000000000100

[20] Woodiwiss AJ , Molebatsi N , Maseko MJ , et al. Nurse‐recorded auscultatory blood pressure at a single visit predicts target organ changes as well as ambulatory blood pressure. J Hypertens. 2009;27:287‐297. 19155786 10.1097/HJH.0b013e328317a78f

[21] Redelinghuys M , Norton GR , Scott L , et al. Relationship between urinary salt excretion and pulse pressure and central aortic hemodynamics independent of steady state pressure in the general population. Hypertension. 2010;56:584‐590. 20733090 10.1161/HYPERTENSIONAHA.110.156323

[22] Sahn DJ , DeMaria A , Kisslo J , Weyman A . Recommendations regarding quantitation in M‐mode echocardiography: results of a survey of echocardiographic measurements. Circulation. 1978;58:1072‐1083. 709763 10.1161/01.cir.58.6.1072

[23] Devereux RB , Alonso DR , Lutas EM , et al. Echocardiograph assessment of left ventricular hypertrophy: comparison to necropsy findings. Am J Cardiol. 1986;57:450‐458. 2936235 10.1016/0002-9149(86)90771-x

[24] Nagueh SF , Smiseth OA , Appleton CP , et al. Recommendations for the evaluation of left ventricular diastolic function by echocardiography: an update form the American Society of Echocardiography and the European Association of Cardiovascular Imaging. J Am Soc Echocardiogr. 2016;29:277‐314. 27037982 10.1016/j.echo.2016.01.011

