# Supplementary figures and images for: Insulin resistance influences the impact of hypertension on left ventricular diastolic dysfunction in a community sample
Source: Clin Cardiol. 2019 Jan 14;42(2):305–11. doi: 10.1002/clc.23145 (PMC6712340; doi:10.1002/clc.23145)

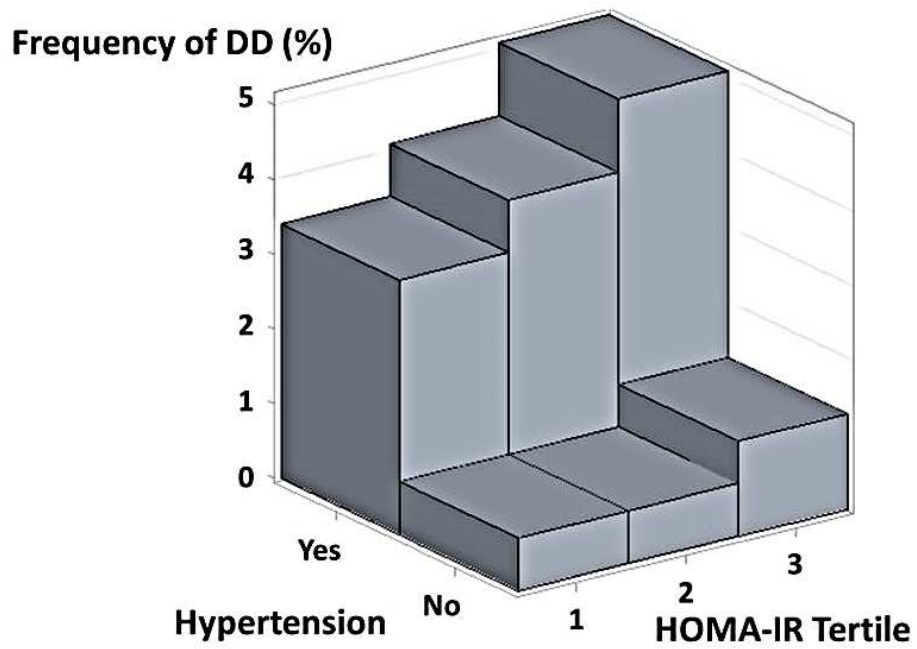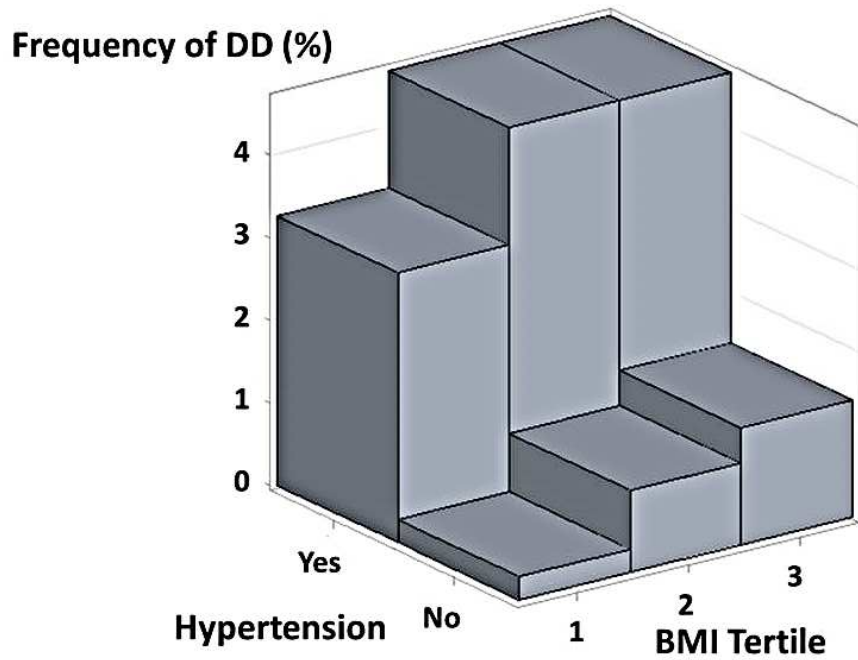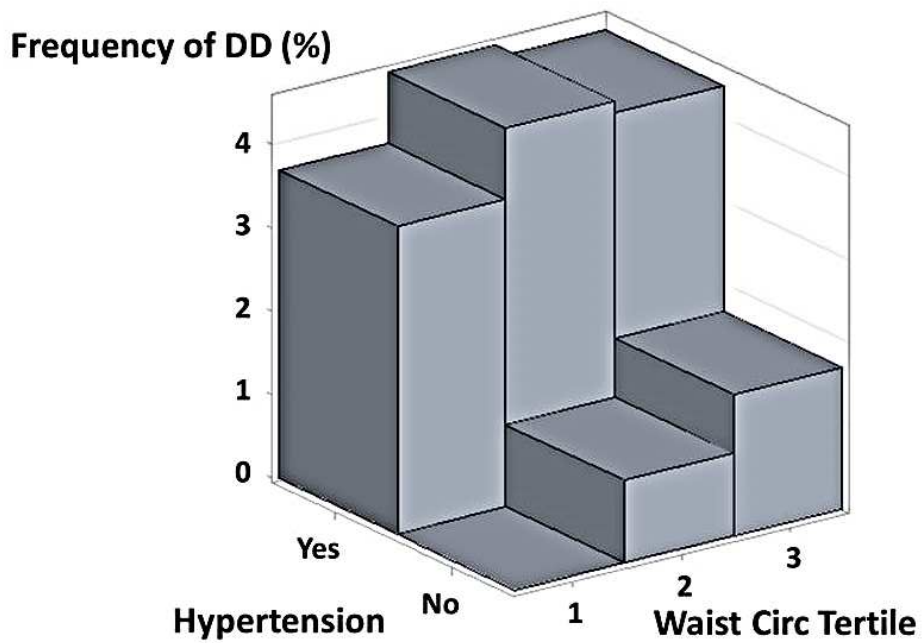

Supplement: Supplementary file 1 — FIGURE S1 Prevalence of left ventricular diastolic dysfunction (DD) in hypertensives as compared to normotensives across tertiles of the homeostasis model of insulin resistance (HOMA‐IR), waist circumference (WC) or body mass index (BMI) in the whole group and across similar age ranges. Tertiles of HOMA‐IR, WC, and BMI are defined in on‐line Table S3. [file CLC-42-305-s001.pdf]
