# Supplementary material for: Insulin resistance influences the impact of hypertension on left ventricular diastolic dysfunction in a community sample
Source: Clin Cardiol. 2019 Jan 14;42(2):305–11. doi: 10.1002/clc.23145 (PMC6712340; doi:10.1002/clc.23145)
Supplement: Supplementary file 2 — TABLE S1 Characteristics of community sample without tissue Doppler imaging (TDI). TABLE S2 Relative contribution (standardized β‐coefficient) of the homeostasis model of insulin resistance, waist circumference or body mass index vs alternative risk factors toward indices of left ventricular diastolic function in a community sample (n = 704). TABLE S3 Ranges of tertiles of the homeostasis model of insulin resistance, waist circumference, and body mass index in normotensives and hypertensives. [file CLC-42-305-s002.docx]

**On-line Supplement**

**Insulin Resistance Influences the Impact of Hypertension on Left Ventricular Diastolic Dysfunction in a Community Sample.**

Adamu J Bamaiyi, Angela J Woodiwiss, Vernice Peterson, Monica Gomes, Carlos D Libhaber, Pinhas Sareli, Gavin R Norton.

Cardiovascular Pathophysiology and Genomics Research Unit, School of Physiology, Faculty of Health Sciences, University of the Witwatersrand, Johannesburg, South Africa.

**Running title**: IR and diastolic function.

**Conflict of interest:** None

AJB, AJW and GRN contributed equally to this work.

This study was supported by the Medical Research Council of South Africa, the University Research Council of the University of the Witwatersrand, and the South African National Research Foundation.

Correspondence and reprint requests: Angela J Woodiwiss: Cardiovascular Pathophysiology and Genomics Research Unit, School of Physiology, University of the Witwatersrand Medical School, 7 York Road, Parktown, 2193, Johannesburg, South Africa. Tel: +27 11 717 2363, e-mail: [angela.woodiwiss@wits.ac.za](mailto:angela.woodiwiss@wits.ac.za)

# FIGURE S1. Prevalence of left ventricular diastolic dysfunction (DD) in hypertensives as compared to normotensives across tertiles of the homeostasis model of insulin resistance (HOMA-IR), WC or BMI in the whole group and across similar age ranges. Tertiles of HOMA-IR, WC and BMI are defined in on-line supplemental table S3.

# TABLE S1. Characteristics of community sample without tissue Doppler imaging (TDI).

#

p value versus with TDI

__________________________________________________________________

Sample number (% female) 340 (65.3) (0.53)

Age (years) 43.5±18.1 0.002

Body mass index (kg/m^2^) 29.1±7.4 0.06

% Overweight/obese/morbidly obese 24.6/20.1/22.5 0.19

Waist circumference (cm) 89.9±15.5 <0.001

% Abnormal waist circumference 42.4 0.007

Regular tobacco (% subjects) 12.6 0.10

Regular alcohol (% subjects) 21.3 0.51

% Diabetes mellitus or an HbA_1c_>6.5% 13.8 0.85

% Treated for hypertension 23.0 0.03

HOMA-IR 3.23±3.28 0.002

Brachial SBP/DBP (mm Hg) 130±23/84±12 0.16/0.23

Left ventricular mass index (g/m^1.7^) 73.0±21.6 <0.0001

__________________________________________________________________

HbA_1c_, glycated hemoglobin; SBP, systolic blood pressure; DBP, diastolic BP; HOMA-IR, homeostasis model of insulin resistance; LV, left ventricle.

**TABLE S2**. Relative contribution (standardized β-coefficient) of the homeostasis model of insulin resistance (HOMA-IR), waist circumference (WC) or body mass index (BMI)versus alternative risk factors toward indices of left ventricular diastolic function in a community sample (n=704).

Models with→ HOMA-IR WC BMI HOMA-IR WC BMI

β-coef±SEM (p-value) β-coef±SEM (p-value) β-coef±SEM (p-value) β-coef±SEM (p value) β-coef±SEM (p value) β-coef±SEM (p value)

______________________________________________________________________________________________________________________

**Log lateral wall e’ Log E/e’**

HOMA-IR -0.103±0.031 (<0.001) - - 0.083±0.036 (0.020) - -

WC - -0.159±0.036 (<0.0001) - - 0.147±0.044 (<0.001) -

BMI - - -0.126±0.034 (<0.0005) - - 0.121±0.040 (0.003)

Age -0.471±0.037 (<0.0001) -0.421±0.041 (<0.0001) -0.447±0.037 (<0.0001) 0.249±0.043 (<0.0001) 0.217±0.050 (<0.0001) 0.226±0.044 (<0.0001)

SBP -0.168±0.033 (<0.0001) -0.152±0.033 (<0.0001) -0.153±0.033 (<0.0001) 0.208±0.038 (<0.0001) 0.188±0.041 (<0.0001) 0.195±0.038 (<0.0001)

Female -0.002±0.033 (0.96) 0.040±0.035 (0.25) 0.049±0.035 (0.17) 0.088±0.039 (0.023) 0.063±0.043 (0.14) 0.043±0.041 (0.30)

**Log septal wall e’ Log LA volume index**

HOMA-IR -0.069±0.029 (0.016) - - 0.040±0.039 (0.30) - -

WC - -0.157±0.038 (<0.0001) - - 0.067±0.047 (=0.16) -

BMI - - 0.110±0.035 (0.002) - - 0.078±0.040 (0.07)

Age -0.495±0.034 (<0.0001) -0.451±0.044 (<0.0001) -0.484±0.038 (<0.0001) 0.187±0.046 (<0.0001) 0.136±0.054 (0.01) 0.162±0.047 (<0.001)

SBP -0.114±0.030 (<0.0005) -0.087±0.035 (0.014) -0.091±0.033 (0.007) 0.107±0.041 (0.010) 0.092±0.043 (0.03) 0.096±0.041 (0.02)

Female -0.003±0.039 (0.91) 0.031±0.037 (0.40) 0.020±0.036 (0.57) -0.083±0.042 (0.05) -0.089±0.046 (0.06) -0.116±0.045 (0.01)

_______________________________________________________________________________________________________________________β-coef, standardized β-coefficient (slope). See table 1 for other abbreviations. Also included in the models are regular tobacco use, and regular alcohol consumption, treatment for hypertension, diabetes mellitus and pulse rate.

**TABLE S3**. Ranges of tertiles of the homeostasis model of insulin resistance (HOMA-IR), waist circumference (WC) and body mass index (BMI) in normotensives and hypertensives.

HOMA-IR WC BMI

____________________________________________________________________________

Normotensives Tertile 1 <0.92 <79 <23.51

Normotensives Tertile 2 0.92 to 2.87 79 to 95 23.51 to 29.86

Normotensives Tertile 3 ≥2.87 ≥95 ≥29.86

Hypertensives Tertile 1 <1.36 <94 <28.26

Hypertensives Tertile 2 1.36 to 6.18 94 to 108 28.26 to 35.55

Hypertensives Tertile 3 ≥6.18 ≥108 ≥35.55

____________________________________________________________________________
